# Reports of Cases

**Published:** 1899-03

**Authors:** 


### REPORTS OF CASES.

### A CASE OF FŒTAL TUBERCULOSIS IN A CALF.[1]

By Mazyck P. Ravenel, M.D.

*(From the Bacteriological Laboratory of the Pennsylvania State Livestock Sanitary Board.)*

Cases of congenital tuberculosis are rare enough to have more than a passing interest; and though this method of transmission occurs so seldom that it may practically be excluded from our calculations in considering the spread of the disease, each undoubted case is instructive and worthy of record. In the human subject the first recorded case is that by Dr. A. Jacobi, of New York, observed in 1861, and reported at the Paris Congress of Tuberculosis in 1891. This is probably the first case observed in man or in the lower animals. Up to the present time, according to Osler (*Practice of Medicine*, third edition), only twenty cases of congenital tuberculosis have been observed in man.

Among the lower animals, if we except experimental work, cases have been seen only in calves, and in them the number has been small. In only seventeen fœtuses have tubercular lesions been found, and in only twelve of these have the tubercle bacilli been present. To these should fairly be added one stillborn calf and seven calves under five days old in which tubercle bacilli were found.

Some authors include a number of other cases, about thirty-eight in all, of calves ranging from seven to fourteen days old, as being cases of congenital tuberculosis; but while not wishing to deny that they are properly classed as such, the proof has seemed to me to be incomplete. While these figures are not complete, they serve to show how seldom congenital transmission of the disease takes place under normal conditions.

The following case has come under my notice: During the latter part of 1898 a cow well advanced in tuberculosis was sent to the Veterinary Department of the University of Pennsylvania. On December 23, 1898, without any assignable reason, she aborted, the fœtus being about seven months old. A careful examination showed it to be normal so far as gross changes were concerned, with the exception of the liver, on which two whitish nodules

[1] Read before the Pathological Society of Philadelphia, February 23, 1899.

about one-sixteenth of an inch in diameter were found. These suggested a tuberculous origin, though much doubt was felt as to their nature. They were crushed in a sterile mortar and emulsified. With the emulsion three guinea-pigs were inoculated—two intraperitoneally and one subcutaneously. One of these pigs died on February 6, 1899, the second February 16th, and the third, inoculated subcutaneously, was chloroformed February 22, 1899. The first two showed general tuberculosis of the abdominal organs with involvement of the inguinal and lumbar glands; the lungs were not involved. The third pig had an abscess at the point of inoculation, about the size of a filbert, filled with caseous material; there was a general tubercular adenitis, with caseous changes in many of the glands; the lungs, liver, spleen, and omentum were markedly tuberculous, and a portion of the peritoneum, about one and one-half inches long by three-fourths of an inch wide, was studded with nodules. This is somewhat unusual, even when a pure culture is injected intraperitoneally. In each of these animals the tubercle bacillus was proven to be present in the lesions.

Unfortunately, I had not the opportunity of examining the placenta of the fœtal calf—a point of much interest and importance. I have since had the opportunity of holding a post-mortem on the mother, and made a careful examination of the uterus, which was free from tuberculous lesions; the disease was confined to the lungs and the lymph-glands of the mesentery.

---

### INVERSION OF THE BLADDER, OR PROLAPSUS VESICÆ, IN THE MARE, FOLLOWING PARTURITION.[1]

By E. J. List, V.S.,
HAVANA, ILL.

*Subject.* A bay mare, weighing about 1400 pounds, well nourished, owned by a farmer, was brought to my barn to be examined by me for a tumor that protruded from the vulva, supposed by the owner to be the uterus.

*History.* She foaled the latter part of April, 1898, at the regular time, bringing forth a dead foal at night, and found by the owner in the morning. She had foaled apparently without trouble. In about three or four days afterward she was stiff, presumably suffering from laminitis. She recovered, however, from this in about three weeks. About this time the owner discovered a tumor

[1] Read before the Illinois State Veterinary Medical Association, February 15, 1899.

protruding from the vagina when lying down, which appeared to increase in size from day to day. This condition was treated lightly by the owner for some time, as it did not inconvenience the animal much except slight straining and dribbling of urine, until he concluded that it was getting worse, protruding from the vagina while standing as well as while lying down. Six weeks had now elapsed since she had foaled.

She was now brought to me to be treated. I prepared to examine her, thinking that I had to deal with an inverted bladder. The tumor at this time presented the size of a well-formed gourd; its base or fundus presented the shape of a gourd, with the exception of its being slightly wrinkled. On my hand entering the vulva I discovered that the tumor protruded from the floor of the vagina at the meatus; posteriorly to this I could feel the cervix uteri in its normal position; thus the diagnosis was clear. Arriving at a proper diagnosis of the condition, I concluded to make an attempt to reduce the viscus to its proper position. This I accomplished, after some time and trouble, by careful manipulations of the thumb and index fingers, assisted by the palm of my hand, slowly at first, until about one-half of the tumor had receded; then the rest followed rapidly. While these manipulations were carried on she strained violently, impeding somewhat the progress of the operation. I now had her moved gently for half an hour, then returned home, with instructions not to allow her to strain. However, in about twenty-four hours it had inverted itself again. This time it was much larger than before, showing signs of patches of inflammation, presumably from the manipulations of the day before. She had strained more or less all the time since the tumor first made its appearance. Now this condition was increased, she straining violently at times. The spontaneous escape of urine became more profuse, making her a disgusting figure to look at.

She was again brought to me for treatment. At the second examination I concluded that a second attempt at reduction would be useless, as the tumor had increased to the size of a man's head, was considerably congested, hot, and small patches presented, presumably the onset of ulceration. While I had used every precaution to prevent these conditions, while replacing it before, by using antispetics and careful manipulations, they were present now, so I concluded to amputate the entire base or fundus of the organ.

*Operation.* There was no preparation of the animal with the exception of antiseptics. I left her standing, placing a twitch on her nose, having an assistant near by. I placed both hands around the viscus, retracted it, bringing it outside the labiæ, having my assistant grasp it and keep it in position. I examined for the openings of the ureters, finding them located at the upper part of the tumor or viscus. I next placed a strong silk ligature around the organ, just posterior to the openings of the ureters, drawing it as tight as possible; next I placed the chain of a Miles écraseur as close to the ligature as possible, tightened it, and removed the base of the organ as rapidly as possible. This caused the animal considerable pain, but was of short duration, as the entire operation consumed only about half an hour. There was scarcely any hemorrhage following the operation. She did not lose an ounce of blood altogether.

After completing the operation I had her moved slowly for half an hour; then returned home, with instructions to inject a solution of bichloride of mercury twice per day. She made an uneventful recovery. No sloughing occurred that was noticed. She continued to dribble urine for some six weeks, but dribbling gradually ceased, she being able to retain part of the urine, and expelling it at intervals until at last she retained it all, apparently expelling it normally.

I had a talk with her owner a few days since. He reports that she is as well as ever, passes urine at intervals apparently normal, is in good flesh, and works every day.

I have reported this case for several reasons: I deem it a very unusual case, because of the accident occurring so long after foaling, and that the bladder had been returned once without success. I believe it all-important that the organ should be amputated immediately when it shows signs of inflammation or cannot be returned. There should be no hesitation. We should never ligate and allow it to slough off, as described by Prof. Fleming in his *Veterinary Obstetrics*. I believe that such operations will surely be followed by inflammation and death of the animal. By removing the bladder with the écraseur immediately we may expect recovery to follow in the majority of cases.

---

Adulteration of Creosote. Upon examination by the Committee on Adulteration of the New York Pharmaceutical Association it was found that fully 50 per cent. of the articles sold in the open market as creosote consisted of carbolic acid, and this was diluted more or less with water, alcohol, and glycerin.

